# P-1997. Real-world Effectiveness of Tixagevimab/cilgavimab for COVID-19 Pre-exposure Prophylaxis in Solid Organ Transplant Recipients during the BA.2.75 and BQ.1 Omicron Variant Period: A matched Case-control Dtudy

**DOI:** 10.1093/ofid/ofae631.2154

**Published:** 2025-01-29

**Authors:** Gun Rojanakit, Sasisopin Kiertiburanakul, Sarinya Boongird, Napan Sutharattanapong, Surasak Kantachuvesiri, Teerapat Yingchoncharoen, Abhasnee Sobhonslidsuk, Jackrapong Bruminhent

**Affiliations:** Faculty of Medicine Ramathibodi Hospital, Mahidol University, Thailand; Faculty of Medicine Ramathibodi Hospital, Bangkok, Krung Thep, Thailand; Faculty of Medicine Ramathibodi Hospital, Mahidol University, Thailand; Faculty of Medicine Ramathibodi Hospital, Mahidol University, Thailand; Faculty of Medicine Ramathibody Hospital, Mahidol University, Bangkok, Krung Thep, Thailand; Faculty of Medicine Ramathibodi Hospital, Mahidol University, Thailand; Faculty of Medicine Ramathibodi Hospital, Mahidol University, Thailand; Faculty of Medicine Ramathibodi Hospital, Mahidol University, Thailand

## Abstract

**Background:**

Solid organ transplant recipients (SOTRs) develop poor immunogenicity after SARS-CoV-2 immunization. The neutralizing monoclonal antibody combination of tixagevimab/cilgavimab (T/C) has been shown to reduce the risk of COVID-19 among this vulnerable population. We assessed the effectiveness and safety of T/C pre-exposure prophylaxis (PrEP) for COVID-19 protection after transplantation.

**Methods:**

We conducted a matched case-control study at a single transplant center during the Omicron lineages BA.2.75 and BQ.1 period (August 2022 to February 2023). T/C doses of 150/150 mg were provided from August 2022 until February 2023, and doses of 300/300 mg were administered thereafter (December 2022 to February 2023). All patients were followed for up to 6 months, and adverse events (AEs) from T/C were assessed.

**Results:**

A total of 143 SOTRs were included, with 115 and 28 SOTRs in the T/C and control groups, respectively. Transplants included kidney (n=108), liver (n=28), and heart (n=9). Over 6 months, 18 (15.7%) in T/C developed COVID-19, with onset at median 3 months. All had mild infections without respiratory failure. Mild and reversible adverse events (AEs) were reported in 25.2% of cases. No rejection occurred. Non-significantly lower COVID-19 within 3 months during BA.2.75 Omicron (8.7% vs. 17.9% in control; p=0.128; effectiveness: 51.4%). At 6 months (BQ.1 Omicron), COVID-19 incidence was 15.7% in T/C vs. 17.9% in control (p=0.667; effectiveness: 12.3%). The occurrence of COVID-19 was not significantly different after adjustment for the number of vaccine doses, history of prior COVID-19, or the dose of T/C.

**Conclusion:**

The effectiveness of T/C tends to be protective against susceptible strains, although the severity of breakthrough infections tends to be mild. Short-term safety as PrEP appears tolerable. TCTR20220801004.

**Disclosures:**

All Authors: No reported disclosures

